# Comparative genomic analysis of *Lacticaseibacillus paracasei* SMN-LBK from koumiss

**DOI:** 10.3389/fmicb.2022.1042117

**Published:** 2022-10-18

**Authors:** Jianghan Wang, Tong Wang, Yandie Li, Zhexin Fan, Zhuoxia Lv, Linting Liu, Xu Li, Baokun Li

**Affiliations:** ^1^School of Food Science and Technology, Key Laboratory of Xinjiang Phytomedicine Resource and Utilization of Ministry of Education, Shihezi University, Shihezi, Xinjiang, China; ^2^Guangdong Yikewei Biotechnology Co., Ltd., Guangzhou, China

**Keywords:** *Lacticaseibacillus paracasei* SMN-LBK, comparative genomics, carbohydrate metabolism, bacteriocin, ethanol tolerance

## Abstract

*Lacticaseibacillus paracasei* SMN-LBK, which was isolated in Xinjiang, has been shown to be a probiotic strain and used as the auxiliary starter for dairy fermentation. Comparative genomic analysis was performed to investigate the metabolic preference and ethanol tolerance mechanisms of *L. paracasei* SMN-LBK. The results of comparative genomics showed that *L. paracasei* strains had high conservation and genetic diversity. SMN-LBK encoded various genes related to carbohydrate and amino acid metabolism pathways, which endow this strain with good fermentation potential. In addition, 6 CRISPR sequences and 8 cas proteins were found in SMN-LBK, and these could play vital roles in the immune system. Furthermore, a unique cluster of potential secondary metabolism genes related to bacteriocins was detected in the genome of SMN-LBK, and this could be important for the preservation of fermented foods. Multiple genes related to alcohol tolerance were also identified. In conclusion, our study explained the traits that were previously demonstrated for SMN-LBK as phenotypes and provided a theoretical basis for the application of SMN-LBK in the food industry.

## Introduction

*Lacticaseibacillus paracasei* is a gram-positive bacterium (Yang et al., 2021) that is usually found in the oral tract, gastrointestinal tract, vagina, fermented foods, and feed (Rothstein et al., 2020; Punia Bangar et al., 2022; Sudhakar and Dharani, 2022); this bacterium has very important functions in the human body, such as its functions in lowering cholesterol and fat levels and its antihypertension and antitumor functions (Erginkaya and Konuray-Altun, 2022; Liu et al., 2022). As a member of an important branch of lactic acid bacteria (LAB), *L. paracasei* is primarily associated with fermented food products (Cuevas-González et al., 2021). *L. paracasei* has a stronger carbohydrate utilization capacity and efficiency than other LAB, which endows it with the ability to adapt to various carbohydrates in different environments (Liu et al., 2011; Cui and Qu, 2021). Some *L. paracasei* strains could also tolerance alcohol and alleviate the alcohol-induced intestinal and alcoholic liver disease (Patel et al., 2021). Microbial genome sequencing is a powerful tool to predict certain important characteristics and reveal metabolic pathways. As of May 2022, 53 *L. paracasei* strains have been sequenced at the whole-genome level. Genomic analysis showed that *L. paracasei* strains have a wide range of sugar utilization patterns and pathways and possess a large number of genes related to carbohydrate metabolism (Cui and Qu, 2021). *L. paracasei* SMN-LBK is a novel probiotic strain that was isolated from koumiss samples collected in Xinjiang. Koumiss is an alcoholic beverage prepared *via* fermentation with LAB and yeast. The alcohol produced by the yeast during the fermentation process imposes alcohol stress on the LAB (Guo et al., 2020). Genes related to the environmental stress response are activated in stressful environments, including genes associated with DNA damage repair, cell wall modification, and heat shock proteins (Galli et al., 2020). Transcriptomic analysis of SMN-LBK treated with alcohol at different concentrations showed that activation of multiple metabolic pathways helps the cells resist ethanol stress and improves the antibiotic of SMN-LBK (Guo et al., 2020), but this phenomenon was not characterized at the gene level.

Comparative genomics is an effective technique to explore the changes in genomic information that occur during interspecies and intraspecies evolution (Wassenaar and Lukjancenko, 2014; Huang D. et al., 2020). Our previous studies showed that SMN-LBK has good fermentation characteristics and probiotic properties and the ability to tolerate ethanol and ameliorate liver injury caused by ethanol in rats, but the underlying mechanism has not been clarified (Guo et al., 2020; Li et al., 2022). Moreover, SMN-LBK has been used in commercial yogurt fermentation as an auxiliary starter, which has extremely high research value and commercial prospects. Comparative analysis of genome sequences could provide information about their fermentation profiles and could be an effective method for elucidating the adaptation of the species to specific environments; this method has been used for *L. plantarum* NCU116 and *L. fermentum* IMDO 130101 (Huang T. et al., 2020; Verce et al., 2020). This systematic based on a comparative analysis of 11 Lactobacillus strains provides an in-depth understanding of their genetic information, evolutionary diversity and metabolic characteristics at the gene level and will play an important role in further understanding the molecular mechanisms related to the fermentation properties and ethanol tolerance of SMN-LBK.

## Materials and methods

### Strains involved in the comparison

*Lacticaseibacillus paracasei* Zhang, which was isolated from koumiss, was the first sequenced *L. paracasei* strain, and its genome sequence has most frequently been used as a reference genome (Wang et al., 2009). *L. paracasei* BD-II, BL23, and N1115 isolated from fermented dairy products (Maze et al., 2010; Ai et al., 2011; Wang et al., 2014) and *L. paracasei* LOCK919 isolated from human feces have all been used as probiotic LAB strains (Koryszewska-Baginska et al., 2013). *L. paracasei* ATCC334 is used as the type strain of *L. paracasei*, and *L. paracasei* W56 has immunomodulatory activity against human dendritic cells (Hochwind et al., 2012; Desfossés-Foucault et al., 2014). The other three strains used in the present study were isolated from yogurt, kimchi, and silage straw (Broadbent et al., 2012; Chen et al., 2012; Sun et al., 2019; Table 1).

**Table 1:** Genomic informations used in the comparative genomic analysis collected from public databases.

Isolation	Genome size (Mb)	GC%	CDSs	Source	Accession number
*L. paracasei* SMN-LBK	3.151087	46.96	2,703	koumiss	CP101831.1
*L. paracasei* ATCC334	2.924330	46.56	2,608	missing	CP000423.1
*L. paracasei* BD-II	3.127290	46.25	2,922	koumiss	CP002618.1
*L. paracasei* BL23	3.079200	46.30	2,884	cheese	FM177140.1
*L. paracasei* LOCK919	3.143370	46.18	2,928	child feces	CP005486.1
*L. paracasei* N1115	3.064280	46.46	2,798	cheese	CP007122.1
*L. paracasei* W56	3.132100	46.25	2,843	missing	HE970764.1
*L. paracasei* Zhang	2.898460	46.42	2,631	koumiss	CP001084.2
*L. casei* 12A	2.907890	46.40	2,669	silage straw	CP006690.1
*L. rhamnosus* GG	3.010110	46.70	2,703	yogurt	CP031290.1
*L. plantarum* ST-III	3.307940	44.50	2,995	Kimchi	CP002222.1

### Gene function prediction

Diamond software (Buchfink et al., 2021) was used to align the amino acid sequence of *L. paracasei* SMN-LBK against the Kyoto Encyclopedia of Genes and Genomes (KEGG; Kanehisa et al., 2019) and Carbohydrate-Active enZYme (CAZy; Lombard et al., 2014) databases. CRISPRdigger (Ge et al., 2016) and the antiSMASH-4.0.2 program (Medema et al., 2011) were used to predict the CRISPR and secondary metabolic gene clusters. R 4.1.2 was used to create carbohydrate heatmaps.

### Phylogenetic tree construction

The single-copy core genes were identified by core−/pan genome analysis, and MUSCLE software (Edgar, 2004) was used for multiple protein sequence alignment. The phylogenetic tree was constructed with TreeBeST software (Vilella et al., 2009) using the neighbor-joining (NJ) method (Naruya and Masatoshi, 1987).

### Comparative genomic analysis

The comparative genomic analysis included analysis of the average nucleotide identity (ANI), gene families, collinearity, and core−/pan genome. Pairwise ANI analysis was performed using fastANI (Jain et al., 2017). Gene family clustering was performed by using BLAST software to perform pairwise alignment of the sequences of proteins encoded in the target genome, and Hcluster-sg (Xu et al., 2021) was used to align the protein sequences according to the alignment similarity with the clustering results. MUMmer software^1^ was used to determine collinearity between target and reference genomes. LASTZ^2^ was used to align the regions to confirm the local positional arrangement relationships and to find the regions of translocation, inversion, and translocation+inversion. CD-HIT software (Li and Godzik, 2006) was used to cluster multiple protein sequences of interest, and then the core−/pan genome results were obtained.

## Results

### Whole-genome phylogenetic analysis

A phylogenetic tree of 15 different species of LAB was constructed for evolutionary analysis based on the core genome (Supplementary Figure 1). The results showed that SMN-LBK was grouped with *L. paracasei* strains. Seven *L. paracasei* strains (ATCC334, BD-II, BL23, LOCK919, N1115, W56, and Zhang), *Lacticaseibacillus casei* 12A, Lacticaseibacillus rhamnosus GG, and *Lactiplantibacillus plantarum* ST-III were selected for the comparative analysis (Table 1). The strains examined here had relatively modest-sized genomes, with various predicted coding sequences, and therefore, the enriched functions could be inferred. In addition, the G + C content of the 10 genomes was relatively moderate, making them more suitable for comparative genomic research; thus, all the selected strains were suitable for comparative analysis.

To assess genetic distance, the ANI values of the genomes were assessed based on orthologous protein-coding genes. The ANI heatmap showed that all the strains were grouped into three distinct clusters (Supplementary Figure 2). The ANI values of the 8 *L. paracasei* strains ranged from 98.0739 to 99.9779% (>95%), suggesting a close evolutionary relationship.

### Gene family analysis

The 29,393 orthologous genes of SMN-LBK and the 10 LAB strains were clustered into 2,494 gene families (Supplementary Table 1). Among the 2,494 gene families, 2,029 gene families were present in SMN-LBK. The number of gene families in the other 7 *L. paracasei* strains ranged from 1,921 to 2,135, similar to that in SMN-LBK, while only 1,346 gene families were found in *L. plantarum* ST-III, which may be due to a distant relationship with *L. paracasei* strains. The numbers of gene families in *L. casei* 12A and *L. rhamnosus* GG were 1,964 and 1,816, respectively, similar to that in the *L. paracasei* strains, further indicating that the number of gene families was affected by genetic distance, which is also consistent with the previous whole-genome phylogenetic analysis and ANI results.

**Figure 1:**
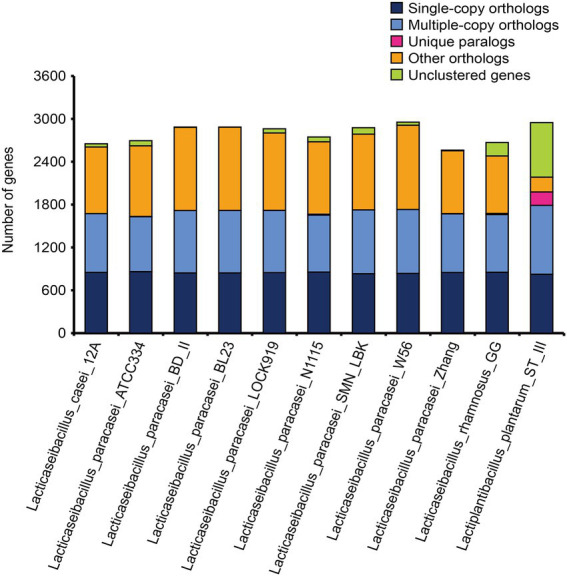
Bar chart of orthologs and paralogs in each strain.

To gain insight into the evolutionary relationships of SMN-LBK, orthologs and paralogs in each strain were analyzed (Figure 1). Among the 1,112 shared gene families, there was no significant difference in the number of single-copy homologous genes and multicopy homologous genes present in the different LAB strains, with values ranging from 824 to 861 (*L. paracasei* SMN-LBK contained 832) and from 769 to 965 (*L. paracasei* SMN-LBK contains 894), respectively.

### Collinearity analysis

**Figure 2:**
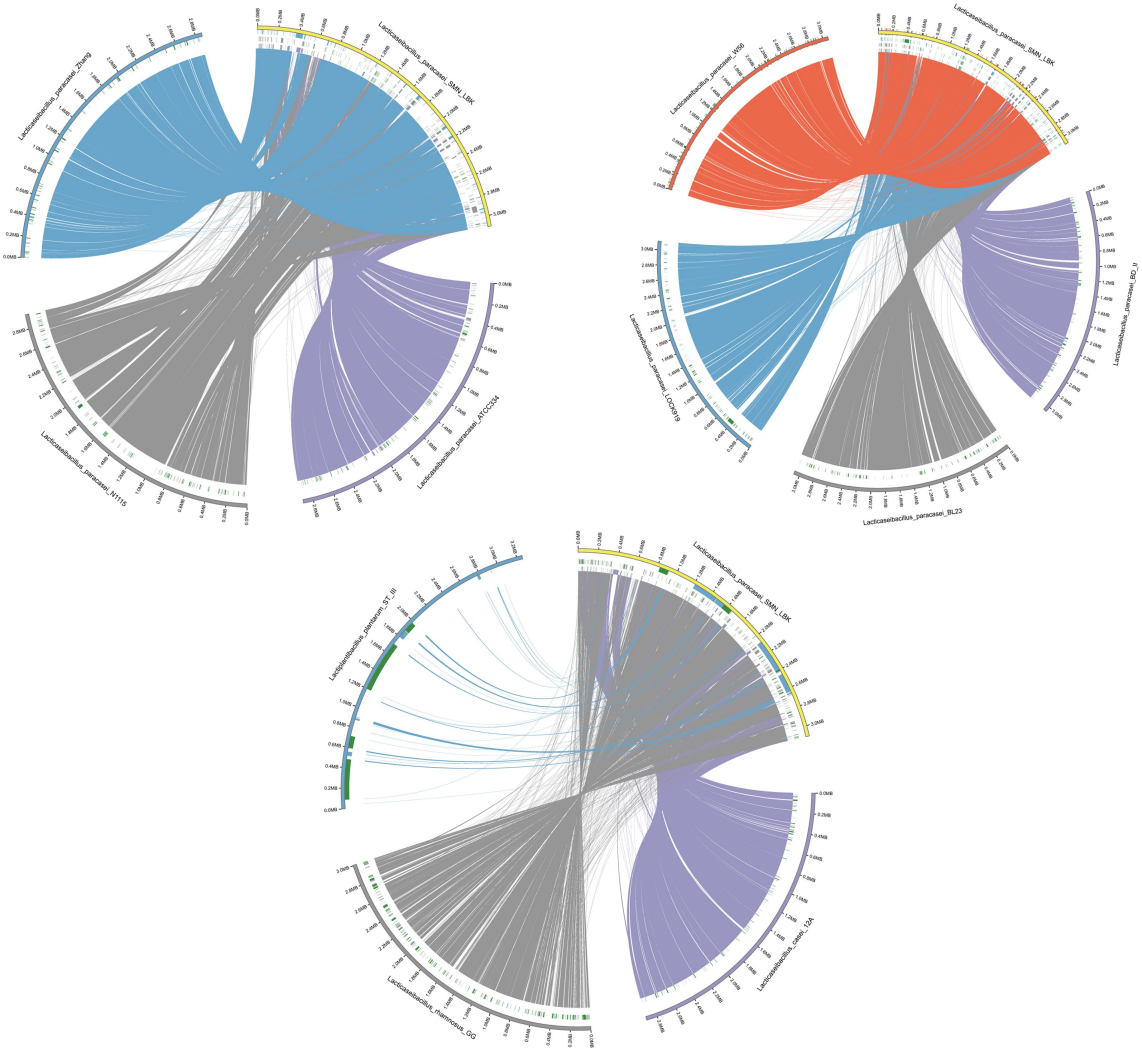
Circos collinearity plot of *L. paracasei* SMN-LBK and ATCC334, BD-II, BL23, W56, Zhang, LOCK919, and N1115. Different colors in the outer circle represent different samples, the reference genome is marked in yellow, the left side is the sample information, and the scale represents the genome scale. The color of the inner circle is the same as that of the outer circle, and the genomes of other strains are connected with the reference genome by lines of corresponding colors. Deletion of the genomes of other strains relative to the reference genome is shown in blocks of corresponding colors. All green blocks indicate insertions in the sample relative to the reference genome (only insertion-deletions longer than 10,000 bp are indicated).

The collinearity of the chromosomes was analyzed to explore the evolution of SMN-LBK. The results showed that the homology between the *L. paracasei* strains and SMN-LBK was relatively high (Figure 2) and that the *L. paracasei* strains had relatively few mutations and recombinations and were genetically stable compared to the other species. The Circos collinearity plot of ST-III and SMN-LBK had large insertions, indicating low homology, which was consistent with the phylogenetic tree and ANI analysis. A considerable number of collinear orthologous genes were identified in the three non-*L. paracasei* strains, indicating conservation among different species. In addition, a visualization study of the target and reference genomes was performed to determine collinearity (Supplementary Figure 3). Only a few insertions or inversions were detected, illustrating that SMN-LBK maintained a good linear relationship with the other 7 strains of *L. paracasei*, which indicated that SMN-LBK had undergone a small number of gene recombination and transformation events during evolution in koumiss. A large number of translocations and inversions were found at 1–585172 bp and 2,210,913–2,938,059 bp in the *L. paracasei* N1115 genome compared with those in SMN-LBK. Species from *L. paracasei* strains showed overall conservation because few rearrangements were found except for N1115. These results are consistent with the results of the phylogenetic tree analysis.

### Core- and pan genome analyses

**Figure 3:**
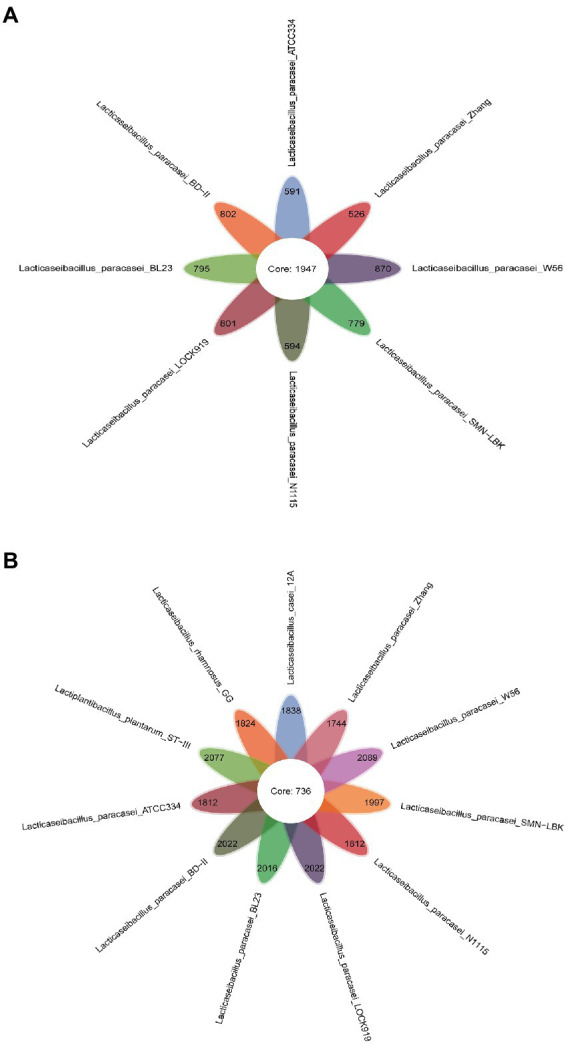
Petaloid diagrams of core and specific genes in *L. paracasei* strains **(A)** and all strains **(B)**. The numbers in the center of the petals represent the number of shared genes; the numbers at the top of the petals represent the number of unique genes.

Core- and pan genome analyses were performed for eight (only *L. paracasei* strains) and 11 (all strains used in this study) LAB strains. A total of 1947 highly homologous genes among all the *L. paracasei* strains were classified as the core genome (Figure 3A), and the number sharply decreased to 736 when the comparison was extended to 11 genomes (Figure 3B), indicating the diversity among the strains at the genome level. In other words, the genomes of the three non-*L. paracasei* strains (*L. casei* 12A, *L. rhamnosus* GG and *L. plantarum* ST-III) were different from those of the *L. paracasei* strains. The core genome is responsible for phenotypic features and basic biological functions (Huang D. et al., 2020). Most core genes of SMN-LBK were necessary for nucleotide, amino acid, ATP and H+ transport and metabolism (GM000641, GM001727, GM002796, GM000481, and GM002358). The pan genome showed the opposite trend compared to the core genome (Supplementary Figure 4). When the number of *L. paracasei* strains was increased, the number of the pan genome increased, indicating the genetic diversity of the *L. paracasei* strains. SMN-LBK seemed to harbor a richer genome because the numbers of unique genes was higher. A total of 215 specific genes were identified in SMN-LBK, 96 of which were annotated with biological functions by KEGG pathway analysis (Supplementary Table 2).

### CRISPR-Cas systems

Six CRISPR sequences were found in SMN-LBK, which were located at 674911–676831 bp, 968,866–969,152 bp, 1,418,571–1,418,983 bp, 1,547,158–1,547,437 bp, 1,808,918–1,809,208 bp, and 2,320,626–2,320,916 bp (Supplementary Table 3). Eight genes encoding cas proteins were also found in SMN-LBK, including 2 Cas1 (GM000687 and GM000692), 2 Cas2 (GM000688 and GM000693), 2 Cas3 (GM000686 and GM000691), 1 Cas4 (GM000689) and 1 Cas5 (GM000690) protein (Supplementary Table 3). All CRISPR-Cas systems include Cas1 and Cas2 proteins that acquire new spacers from invasive elements.

### Carbohydrate metabolism and amino acid metabolism

**Figure 4:**
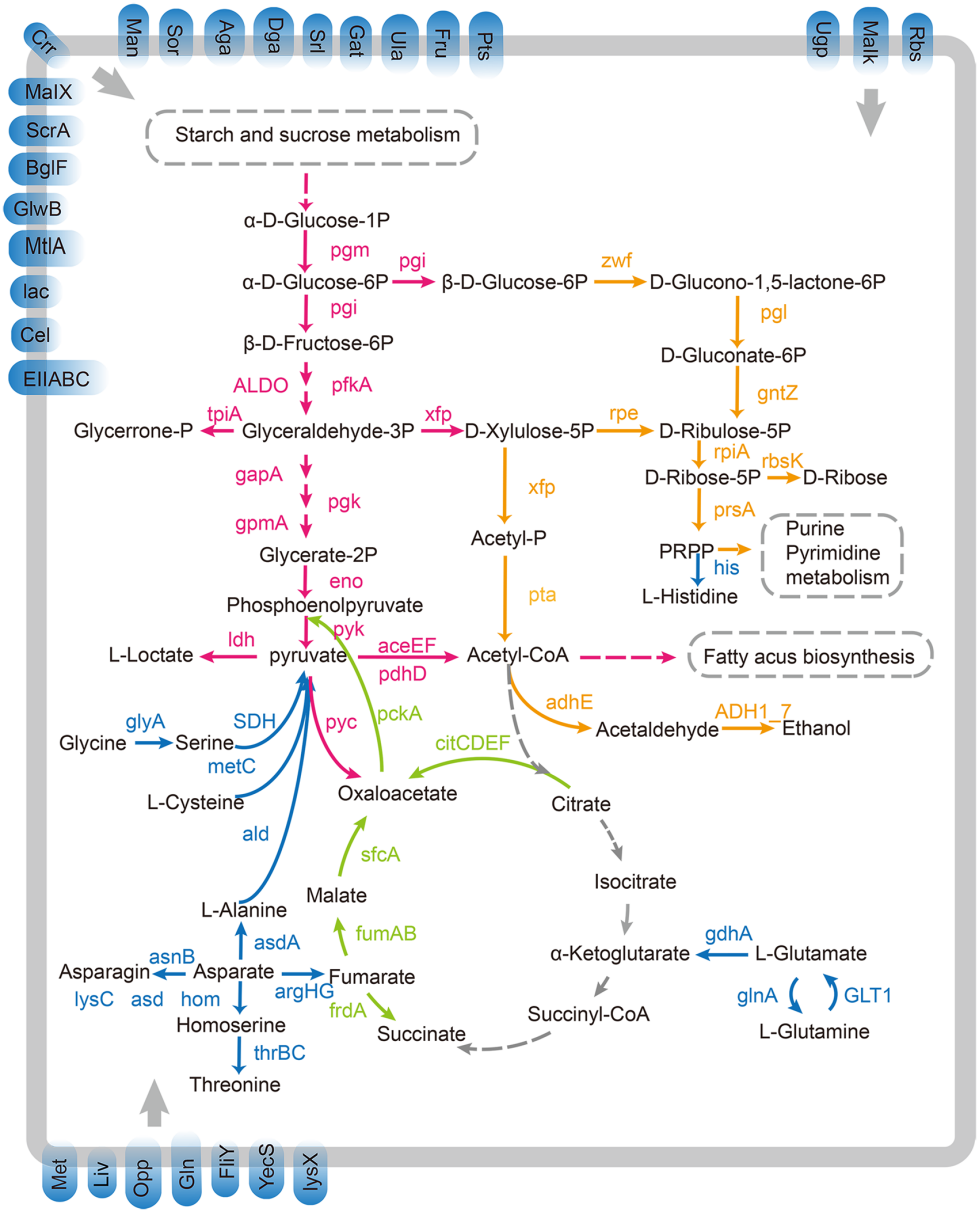
Overview of proposed pathways present in *L. paracasei* SMN-LBK. Pink arrows represent the homolactic fermentation pathway. Orange arrows represent the heterolactic fermentation pathways. Green arrows represent the incomplete citrate metabolism pathways. Blue arrows represent the proteolysis and amino acid metabolism pathways. Blue squares embedded in the membrane represent PTSs and ABC transporters. Gray dashed arrows represent the absence of this pathway. Gray dashed boxes represent other metabolic pathways.

The number of carbohydrate-binding modules (CBMs), carbohydrate esterases (CEs), glycoside hydrolases (GHs), glycosyltransferases (GTs), and polysaccharide lyases (PLs) genes in *L. paracasei* SMN-LBK was similar to that in the other 7 *L. paracasei* strains, and none of the strains encoded auxiliary activities (AAs; Supplementary Figure 5 and Supplementary Table 4). In addition, 1–3 PL-related genes were found in only the 8 *L. paracasei* strains and *L. casei* 12A but not in *L. rhamnosus* GG and *L. plantarum* ST-III, showing that the *L. paracasei* strains and *L. casei* 12A had a stronger ability to hydrolyze and utilize polysaccharides. *L. paracasei* SMN-LBK had potential genes encoding various carbohydrate metabolism and amino acid metabolism pathway-related enzymes. Genes involved in carbohydrate and amino acid transport and decomposition were used to reconstitute the metabolic pathways of *L. paracasei* SMN-LBK (Figure 4). The numbers of genes involved in the glycolysis pathway, the tricarboxylic acid (TCA) cycle and the pentose phosphate pathway were roughly similar among the 11 strains (Supplementary Table 5). In addition, SMN-LBK encodes a complete proteolytic system and pathways for the transformation and metabolism of multiple amino acids, suggesting its potential ability to use proteins as substrates for fermentation.

### Potential to produce bacteriocin and alcohol tolerance

**Figure 5:**
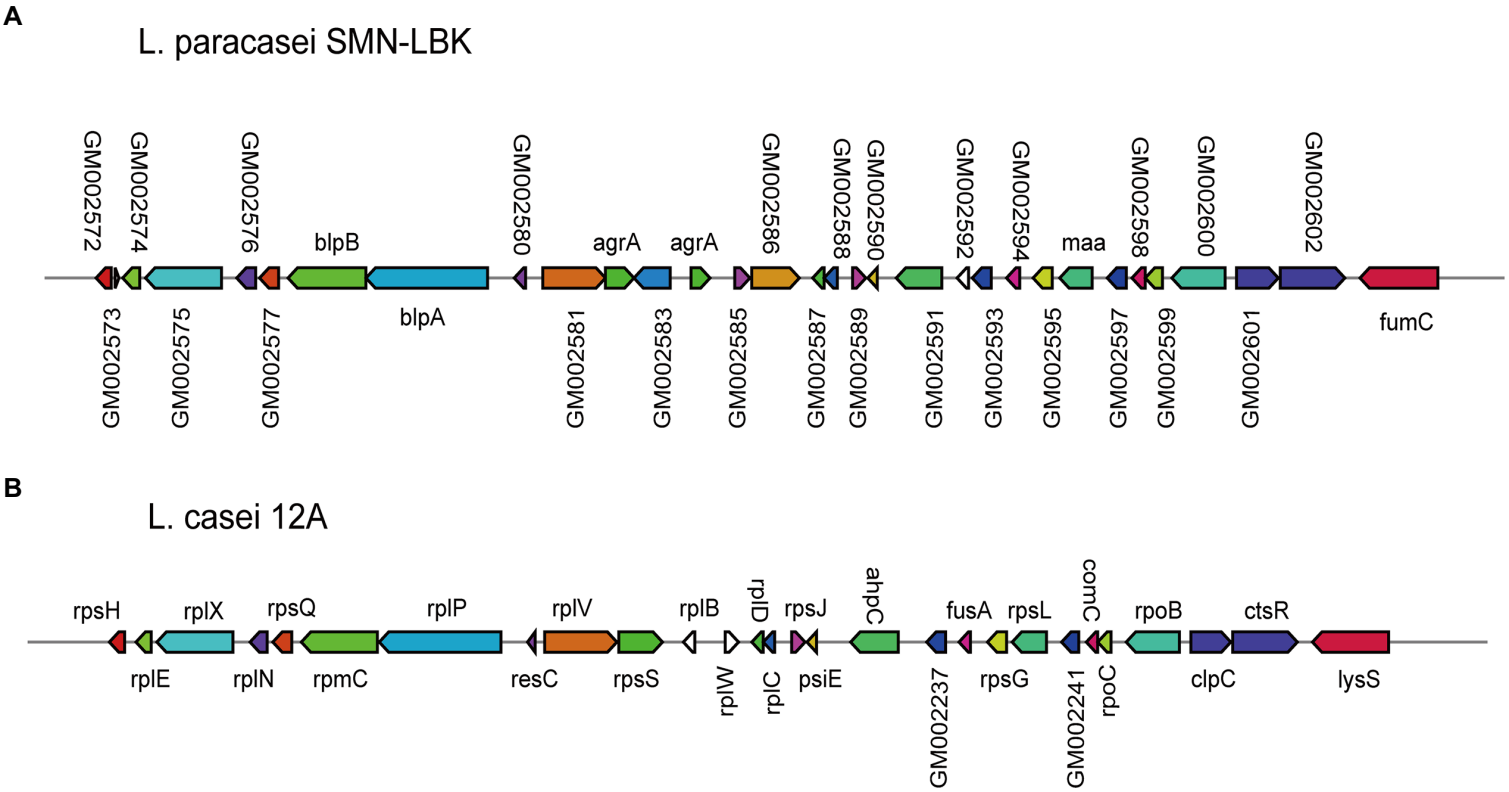
Comparative analysis of bacteriocin-producing loci for *L. paracasei* SMN-LBK and *L. casei* 12A. **(A)** Represents the bacteriocin produced by *L. paracasei* SMN-LBK. **(B)** Represents the bacteriocin produced by *L. casei* 12A. Different colors and sizes represent different genes.

To date, research on *L. paracasei* strains has mainly focused on its fermentative products, while research on the production of bacteriocins has been relatively limited. *L. paracasei* SMN-LBK encoded a bacteriocin secondary metabolism gene cluster (Figure 5). The gene cluster of SMN-LBK (from GM002572 to GM002603) was 86% similar to that of 12A, while the other 9 strains did not contain this gene cluster. Eleven LAB strains encoded various genes related to alcohol tolerance (Supplementary Table 6), which were related to key enzymes associated with glucose metabolism, heat shock proteins, DNA damage repair, and oxidative stress response proteins.

## Discussion

### Comparative genomic analysis

Whole-genome phylogenetic analysis, ANI values, gene family, collinearity analysis, core- and pan genome analysis are general genomic contents that reflect the bacterial genome evolution. The ANI values of the 8 *L. paracasei* strains isolated from different sources were not significantly different, suggesting that there was no correlation between the strain clustering and niches. However, as expected, the ANI of *L. plantarum* ST-III was 0%, indicating a large genetic distance. Interestingly, the *L. casei* 12A, *L. rhamnosus* GG, and *L. paracasei* strains were not strains of the same species, but their ANI values compared with the *L. paracasei* strains reached more than 80%, and the ANI values of *L. casei* 12A and *L. paracasei* Zhang reached 99.131%, which may be due to *L. casei*, *L. rhamnosus* and *L. paracasei* belonging to the *Lacticaseibacillus casei* group (LCG; Cui and Qu, 2021). This result is compatible with the genetically closely related LCG (Hill et al., 2018).

It is believed that some *L. paracasei* strains present remarkably strong ability to metabolize various carbohydrate (Stefanovic and McAuliffe, 2018; Cui and Qu, 2021). The results of Core- and pan showed that 20 specific genes were related to glucose metabolism, which may endow SMN-LBK with a stronger capacity for carbohydrate metabolism. Mannose and fructose phosphotransferase systems (PTSs) were found encoded among the unique genes of SMN-LBK, including mannose-specific IIACD components and fructose-specific IIABC components, indicating the ability of SMN-LBK to transport mannose and fructose. The mannose PTS in SMN-LBK acts *via* a mechanism that couples translocation with substrate phosphorylation. Ten genes involved in energy metabolism were also found among the unique genes of SMN-LBK, including genes encoding 6 F-type H + -transporting ATPase subunits, which utilize the energy of the electrochemical H+ gradient generated by electron transfer to catalyze the synthesis of ATP during oxidative phosphorylation. In addition, 8 ABC transporter-related genes were encoded in SMN-LBK. ABC-2-type transport system ATP-binding protein-related genes were also found in SMN-LBK; the encoded proteins can utilize the energy generated by ATP hydrolysis and mediate the transport of various endogenous and exogenous substances (Wang et al., 2021), suggesting that SMN-LBK has a stronger ATP transport capacity. The ability to transport ATP enables SMN-LBK to maintain normal growth in adverse environments.

In prokaryotes, the CRISPR-Cas system confers resistance to foreign plasmids and phage sequences and recognizes and silences invading functional elements, thereby acting as the immune system (Dong et al., 2022; Shin and Kim, 2022). Types I, II, III, IV, V, and VI of CRISPR systems have been reported so far (Wright et al., 2016). A type I–E CRISPR-Cas system was found in ATCC334, and a type II-A CRISPR-Cas system was found in Zhang, BD-II, and LOCK919 (Yang et al., 2020). In each isotype, the repeat lengths were conserved. The lengths of the repeats of the six CRISPR sequences in SMN-LBK were 28, 41, 41, 41, 40, and 41 bp. Therefore, it was speculated that SMN-LBK may contain one incomplete type I-E repeat and five incomplete type II-A repeats based on the fact that type II-A repeats are 36 nucleotides in length and type I-E repeats are 28 nucleotides in length (Hidalgo-Cantabrana et al., 2019), as previously described for other strains. Yang analyzed the CRISPR-Cas systems of 58 *L. paracasei* strains and found that 43% of the *L. paracasei* strains encoded a CRISPR-Cas system, and a majority of the strains harbored type II-A CRISPR-Cas systems (Yang et al., 2020). The type II-A CRISPR-Cas system destroys the double-stranded DNA of invading genetic material and prevents DNA repair to achieve immunity (Bernheim et al., 2017). Therefore, further identification of the CRISPR system is of great importance for obtaining a comprehensive understanding of SMN-LBK.

### Carbohydrate metabolism

Various genes encoding starch and sucrose metabolism proteins in SMN-LBK, such as 1,4-alpha-glucan branching enzyme (glgB), glycogen phosphorylase (glgP), and neopullulanase (nplT), which degrade large-molecule sugars into small-molecule sugars, were found in the *L. paracasei* strains. Macromolecular carbohydrates such as oligosaccharides and starches can be transported into cells *via* consumption of ATP through the PTS and ABC transport systems and then degraded into monosaccharides (such as glucose, fructose, and mannose) by α-amylase (GM001083), 1,4-alpha-glucan branching enzyme (GM002209), and alpha-glucosidase (GM002295) secreted by SMN-LBK. Additionally, a complete mannitol operon, containing a mannitol operon transcriptional antiterminator (mtlR), mannitol-specific IIA component (mtlA), and mannitol-1-phosphate 5-dehydrogenase (mtlD), was encoded by SMN-LBK; mannitol plays important roles in osmoregulation and stress tolerance (Jennings, 1985; Stoop and Mooibroek, 1998). Various specific monosaccharides and disaccharides PTSs have been characterized in *L. paracasei via* the sugar consumption analysis (Veyrat et al., 1994). 99 genes related to PTSs were found in *L. paracasei* SMN-LBK, and 30 genes were associated with fructose and mannose metabolism. The numbers of genes associated with PTSs in SMN-LBK, BL23, LOCK919, and W56 were roughly similar, all exceeding 100, while the remaining three strains of *L. paracasei* had fewer genes related to PTSs. Nutrients such as extracellular carbohydrates are mainly transported into cells through PTSs (Jeckelmann and Erni, 2020). PTSs transfer phosphate groups and nutrients such as extracellular carbohydrates into the intracellular space during sugar uptake and metabolism (Galinier and Deutscher, 2017).

The extensive studies have indicated that *L. paracasei* strains possess various sugar metabolism pathways (Wu and Shah, 2017). The genome of SMN-LBK contained genes encoding key enzymes involved in the glycolysis and pentose phosphate pathways (Figure 4), which are the main metabolic pathways for homolactic fermentation and heterolactic fermentation, respectively. Lactic acid fermentation is a hallmark metabolic process of LAB strains (Huang T. et al., 2020). Pyruvate is a common intermediate metabolite of the glycolysis and pentose phosphate pathways and plays an important role in carbohydrate metabolism, amino acid metabolism, and fat metabolism. Lactic acid is mainly formed by the reduction of the carbonyl group of pyruvate under the catalytic action of lactate dehydrogenase (Rao et al., 2021). SMN-LBK harbors genes encoding D-lactate dehydrogenase (ldhA) and L-lactate dehydrogenase (ldh), which catalyze the transfer of H+ from NADH+ to pyruvate, which is then reduced to lactate under oxygen deficiency or anaerobic conditions. The strong lactate synthesis potential of SMN-LBK during milk fermentation may be because it encodes five ldh genes (unpublished). SMN-LBK also encodes various genes related to pyruvate production and conversion. Orthophosphate dikinase (ppdK) in SMN-LBK catalyzes the conversion of pyruvate to phosphoenolpyruvate. Phosphoenolpyruvate is catalytically acted upon by pyruvate kinase (pyk), which transfers the phosphate group originally attached to the oxygen atom to ADP, generating pyruvate and a large amount of ATP. Pyruvate is converted to oxaloacetate, an important intermediate, by the action of pyruvate carboxylase (pyc) in SMN-LBK. The phosphoenolpyruvate-pyruvate-oxaloacetate node is a major branch of central carbon metabolism and serves as a junction for glycolysis, gluconeogenesis, and the TCA cycle (Llamas-Ramírez et al., 2020). In addition, pyruvate oxidase (poxL), acetate kinase (ackA), and acyl phosphatase (acyP) catalyze the conversion of pyruvate to ATP and acetate, which is one of the main sources of the sour taste in fermented dairy products. Pyruvate can also be converted to acetyl-CoA by the pyruvate dehydrogenase complex, consisting of pyruvate dehydrogenase component (aceEF), dihydrolipoyl transacetylase, and dihydrolipoate dehydrogenase (pdhD). Acetyl-CoA is an important intermediate metabolite for energy substances, and it links the metabolic pathways of energy substances in the body, such as carbohydrate, amino acid and fat metabolism, including the TCA cycle and oxidative phosphorylation pathway (Shi and Tu, 2015). SMN-LBK encodes acetaldehyde dehydrogenase (adhA) to convert acetyl-CoA to acetaldehyde, which is the main volatile flavor compound in fermented dairy products, and aldehydes can be used for the biosynthesis of other flavor compounds (Xiao et al., 2018). SMN-LBK encodes an incomplete TCA cycle that cannot complete the metabolism to CO_2_, as in eukaryotes (Figure 4). Oxaloacetate, malate, fumarate and succinate are interconverted in the incomplete TCA system. Oxaloacetate is a very important intermediate product that is mainly synthesized by pyruvate carboxylation, which is catalyzed by pyc in the metabolic pathway and can also be catalyzed by citrate lyase (citCDEF). Malate is produced from pyruvate by malate dehydrogenase (sfcA) and then finally converted to succinate by fumarate hydratase (fumA, fumB) and fumarate reductase flavoprotein subunit (frdA). The presence of this elaborate carbohydrate uptake and degradation machinery suggests that SMN-LBK may thrive and be predominant during dairy fermentation. However, some putative carbohydrate utilization related genes need further study to determine their functions.

### Proteolysis and amino acid metabolism

Bacteria with the ability to degrade casein to peptides and amino acids can meet their growth requirements for nitrogen sources (Song et al., 2016). Proteins are degraded to oligopeptides by extracellular proteases, and the oligopeptides are then transported into cells through a specific peptide transport system. The peptides transported into the cell by the transport system are further degraded into smaller peptides or amino acids by various intracellular peptidases (Juillard et al., 2022). Several genes related to cellular envelope proteases were found in SMN-LBK (Supplementary Table 5), such as scpA (GM001565) and scpB (GM001566). SMN-LBK also encodes two types of peptide transport systems, including two complete Opp operons (oppABCDF), three separate oligopeptide transport system substrate-binding proteins (oppA), and one dipeptide transport system permease protein (dppC; Figure 4). Interestingly, except for BD-II, BL23, and W56, the other 7 LAB strains did not encode the Dpp transport system. In addition, genes encoding the branched-chain amino acid transport system substrate-binding protein (livFHKM) were found; these genes would enable SMN-LBK to capture amino acids from the fermentation environment for nitrogen metabolism. Various genes related to oligopeptidase activity were found in SMN-LBK, including those encoding dipeptidase (pepDA, pepDB), oligoendopeptidase (pepF), Xaa-Pro aminopeptidase (pepP), dipeptide aminopeptidase (pepT), aminopeptidase (pepNS), Xaa-Pro dipeptidase (pepQX), endopeptidase (pepO) and bleomycin hydrolase (pepC). Xaa-Pro dipeptidase is a proline-specific protease that plays an important role in industrial applications in cheese ripening by cleaving the proline-rich sequence of beta-casein. Overall, the genome of SMN-LBK encodes a large number of proteases and peptidases that form a complete proteolytic system, which may endow SMN-LBK with strong proteolytic ability.

Eleven strains in this study encoded a transcriptional regulator of arginine metabolism (argR) and an arginine succinate metabolism operon composed of an argininosuccinate synthase (argG) and an argininosuccinate lyase (argH), the reaction processes of which are related to the coercion response (Figure 4). ArgR has been reported to play an important role in the regulation of arginine metabolism in LAB strains (Cunin et al., 1983). It has been demonstrated that upregulation of argG and argH gene expression was associated with the acid stress response in *L. paracasei* strains (Wu et al., 2011). ArgG and argH catalyze the conversion of aspartate to fumarate, which then enters the TCA cycle for oxidation. L-glutamine is converted to L-glutamate by glutamate synthase (GLT1), and the process is accompanied by the generation of NADH, which is finally converted to 2-oxoglutarate by glutamate dehydrogenase (gdhA); 2-oxoglutarate enters the TCA cycle to provide energy for the body. At the same time, 2-oxoglutarate can be used to regenerate L-glutamine by GLTI and glutamine synthetase (glnA), and the L-glutamine is then converted to carbamoyl-phosphate by carbamoyl-phosphate synthase (CAD). Carbamoyl-phosphate then enters arginine biosynthesis and pyrimidine metabolism. Moreover, L-serine, glycine and L-cysteine are catalytically converted by L-threonine ammonia-lyase (SDH), hydroxymethyltransferase (glyA) and cysteine-S-conjugate beta-lyase (metC), respectively, to pyruvate, which enters the TCA cycle and pyruvate metabolism, linking amino acid metabolism and sugar metabolism. The genes in SMN-LBK also encode a phosphoribosyl-related enzyme (hisABCDEFGHI) that catalyzes the conversion of PRPP to L-histidine. SMN-LBK also encodes asnB, which converts asparate to asparagine. It has been proposed that the conversion of amino acids and aminoacyl generates ammonia, so various aminoacylases, including asparagine synthase (asnB), play an important role in maintaining the intracellular pH balance to allow the cells to resist the acidic stress generated during fermentation and metabolism (Champomier Verges et al., 1999). SMN-LBK encodes the biosynthesis-related genes of various amino acids, which provide a special flavor to fermented dairy products (Widyastuti and Febrisiantosa, 2014).

### Potential to produce bacteriocin and alcohol tolerance

The extensive studies have indicated that *L. paracasei* can produce bacteriocin, a bacteriostatic active substance, which can inhibit many spoilage bacteria and pathogenic bacteria in food (Qi et al., 2021; Ye et al., 2021). A bacteriocin secondary metabolism gene cluster was identified in SMN-LNK that was composed of 32 genes, but only 6 genes (blpA, blpB, agrA, maa and fumC) were annotated. BlpA and blpB, which are bacteriocin exporters, enable SMN-LBK to export bacteriocins. The LytTR family-related genes (agrA) of SMN-LBK are response regulators that control the synthesis of virulence factors and other exoproteins. The agrA response regulator is also an accessory gene regulator protein A (agrA) in Staphylococcus aureus, which is a member of two-component regulatory systems (Rajasree et al., 2016). Traditional antibiotics can accelerate the development of resistance in microorganisms, making various pathogens highly resistant to antibiotics. An inhibitor developed on the basis of the AgrA/C two-component signaling system could reduce infection by pathogenic microorganisms without the development of resistance, which is the next frontier in the innovative development of modern antibacterial drugs (Palaniappan et al., 2021). These properties allow SMN-LBK to thrive and endow it with the potential to inhibit the growth of undesirable microorganisms in fermented dairy products.

For most species of LAB, alcohol tolerance is closely related to key enzymes associated with glucose metabolism, heat shock proteins, DNA damage repair, and oxidative stress response proteins (Luo et al., 2014). Glucose metabolism-related genes, such as 6-phosphofructokinase (PFK), glycerol kinase (GK) and lactate dehydrogenase (ldh), were found in the genome of SMN-LBK. Guo overexpressed PFK and GK in *Lactococcus lactis* NZ9000 and found that the survival rate under 10% ethanol stress was significantly increased (Guo et al., 2020). To resist alcohol stress, SMN-LBK could accelerate the production of ATP by increasing the enzymatic activity of important enzymes in the glycolytic pathway to maintain cell stability. Transcriptional regulators (HrcA, CtsR), a heat stress protein (the chaperonin GroELS), a molecular chaperone (DnaJK) and an ATP-dependent Clp protease (ClpBCELPQX) were encoded in the 11 LAB strains. The CtsR regulon was previously shown to be involved in ethanol stress responses in *L.plantarum* (Van Bokhorst-van de Veen et al., 2011) and *B. subtilis* (Gerth et al., 2002). GroELS and DnaJK were positively regulated by HrcA, ClpBCELPQX was negatively regulated by CtsR, and the transcription of HrcA and CtsR increased and reduced, respectively, under ethanol stress, indicating that GroELS, dnaJK and ClpBCELPQX may be related to ethanol tolerance. Moreover, the oxidative stress protein thioredoxin (trxA) was also significantly upregulated under ethanol stress (Van Bokhorst-van de Veen et al., 2011). High concentrations of ethanol induced DNA damage, such as DNA strand breaks and base pair excision, and then triggered DNA repair (Nagaria et al., 2013). RecA is the most critical protein in the process of DNA homologous recombination repair (Horváth et al., 2008). Helicase (UvrD) can unwind double-stranded DNA for DNA repair by binding and hydrolyzing ATP (Feliciello et al., 2018). When alcohol damages DNA of *L. paracasei*, the recombination protein (RecA) and helicase (UvrD) in SMN-LBK will be activated for DNA repairing to ensure the normal physiological activity in SMN-LBK. Genes related to glucose metabolism, heat stress, oxidative stress and DNA repair play important roles in the stress resistance mechanism of SMN-LBK under ethanol stress. The potential of SMN-LBK to tolerate ethanol was proved at the gene level, but the ethanol tolerance mechanism of SMN-LBK needs to be further explored by designing experiments.

## Conclusion

In this study, comparative genomic analysis provided a better opportunity to understand the metabolic preferences of *L. paracasei* SMN-LBK. The results of phylogenetic tree, ANI, gene family, collinearity and core−/pangenome analyses indicated the genetic diversity and conservation among *L. paracasei* strains. In addition, 6 CRISPR sequences and 8 cas proteins in SMN-LBK were investigated; the CRISPR-Cas system is associated with resistance to invasion by foreign plasmids and phage sequences. SMN-LBK encoded multiple essential genes required for carbohydrate metabolism and protein metabolism, which endowed SMN-LBK with a strong fermentation capacity. A unique bacteriocin-related gene cluster was identified in SMN-LBK, and this cluster was highly homologous with *L. casei* 12A, suggesting that SMN-LBK may play an important role in the preservation of fermented food. Moreover, various genes associated with ethanol stress tolerance were identified, and the potential ethanol tolerance of SMN-LBK was confirmed at the gene level. The detailed and accurate genetic information for the probiotic SMN-LBK will be indispensable for further research on SMN-LBK and of its potential applications in the food industry.

## Data availability statement

The original contributions presented in the study are included in the article/Supplementary material, further inquiries can be directed to the corresponding author.

## Author contributions

JW and BL wrote the manuscript draft, designed the experiments, and performed analyses of data for comparative genomics. JW and TW analyzed the metabolism pathways. ZF supervised lab work. YL, ZL, LL, and XL revised the manuscript. All authors contributed to the article and approved the submitted version.

## Funding

This research was supported by the Natural Science Foundation of China (grant number 32060548) and the Xinjiang Uygur Autonomous Region International Cooperation Project (2022E01028).

## Conflict of interest

XL was employed by Guangdong Yikewei Biotechnology Co., Ltd.The remaining authors declare that the research was conducted in the absence of any commercial or financial relationships that could be construed as a potential conflict of interest.

## Publisher’s note

All claims expressed in this article are solely those of the authors and do not necessarily represent those of their affiliated organizations, or those of the publisher, the editors and the reviewers. Any product that may be evaluated in this article, or claim that may be made by its manufacturer, is not guaranteed or endorsed by the publisher.

## Footnotes

^1^ http://mummer.sourceforge.net/

^2^ https://github.com/lastz/lastz
