# Supplementary figures and images for: Comparative genomic analysis of Lacticaseibacillus paracasei SMN-LBK from koumiss
Source: Front Microbiol. 2022 Oct 18;13:1042117. doi: 10.3389/fmicb.2022.1042117 (PMC9622802; doi:10.3389/fmicb.2022.1042117)

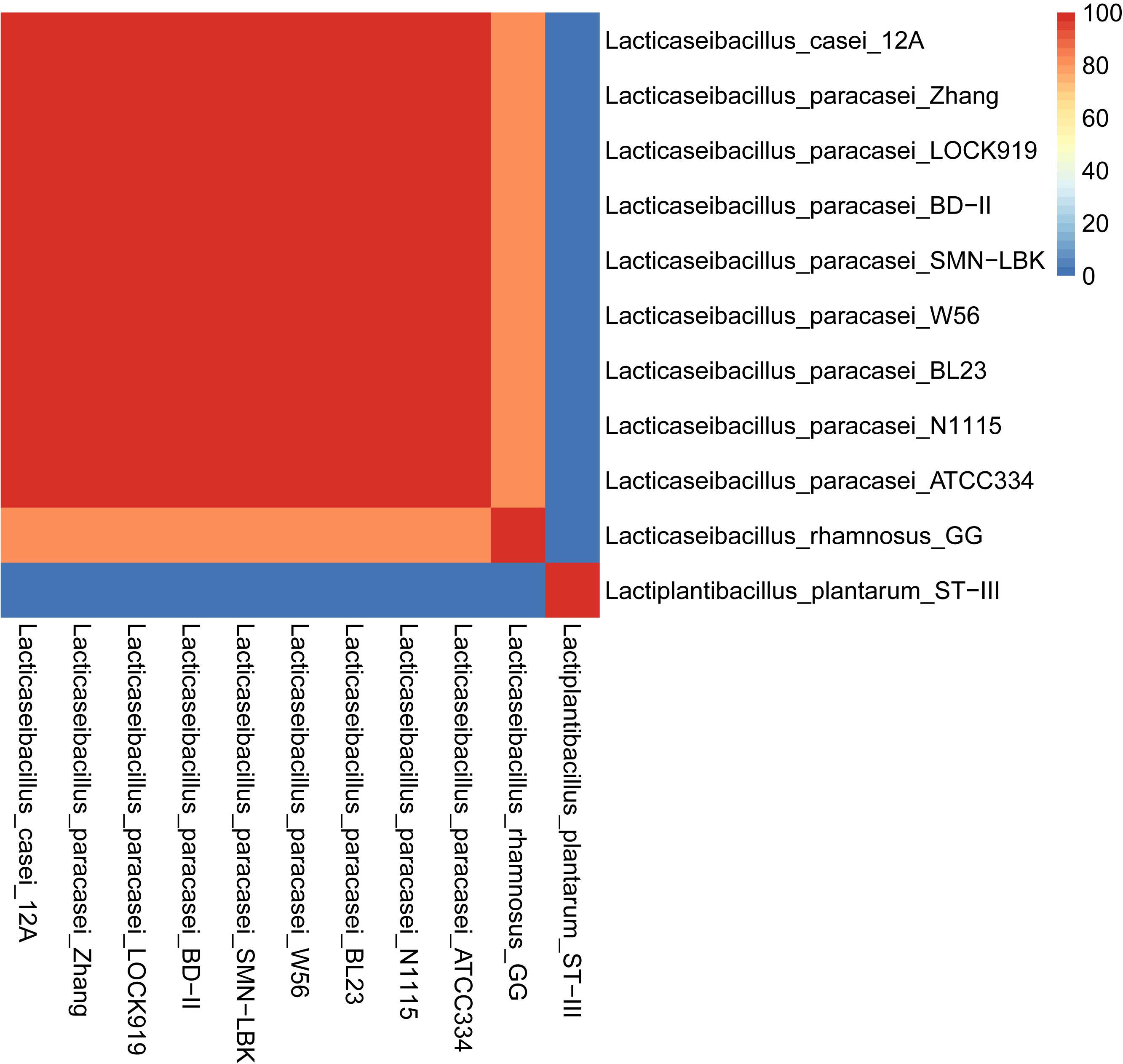

Supplement: Supplementary file 2 [file Image_2.JPEG]

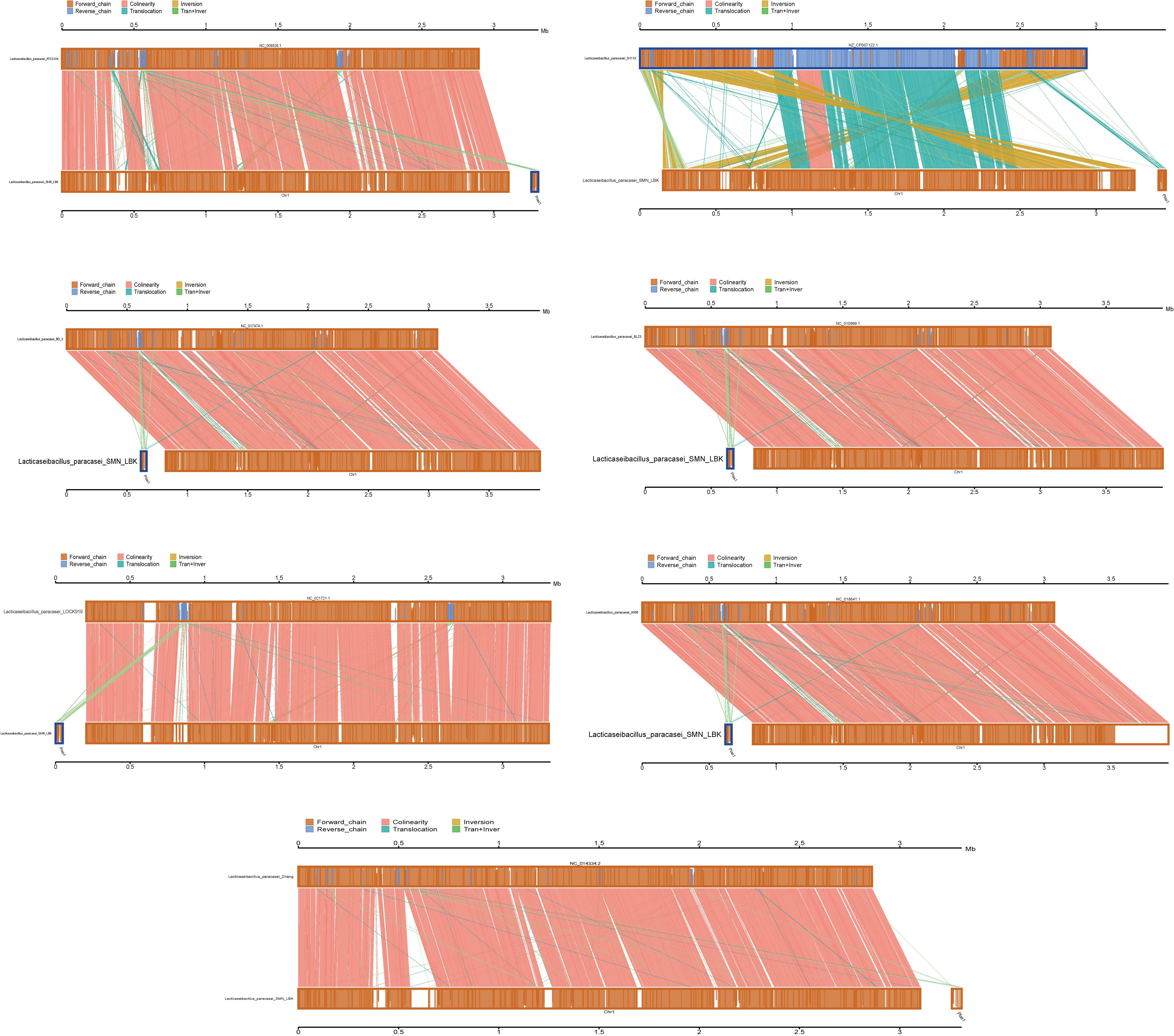

Supplement: Supplementary file 3 [file Image_3.JPEG]

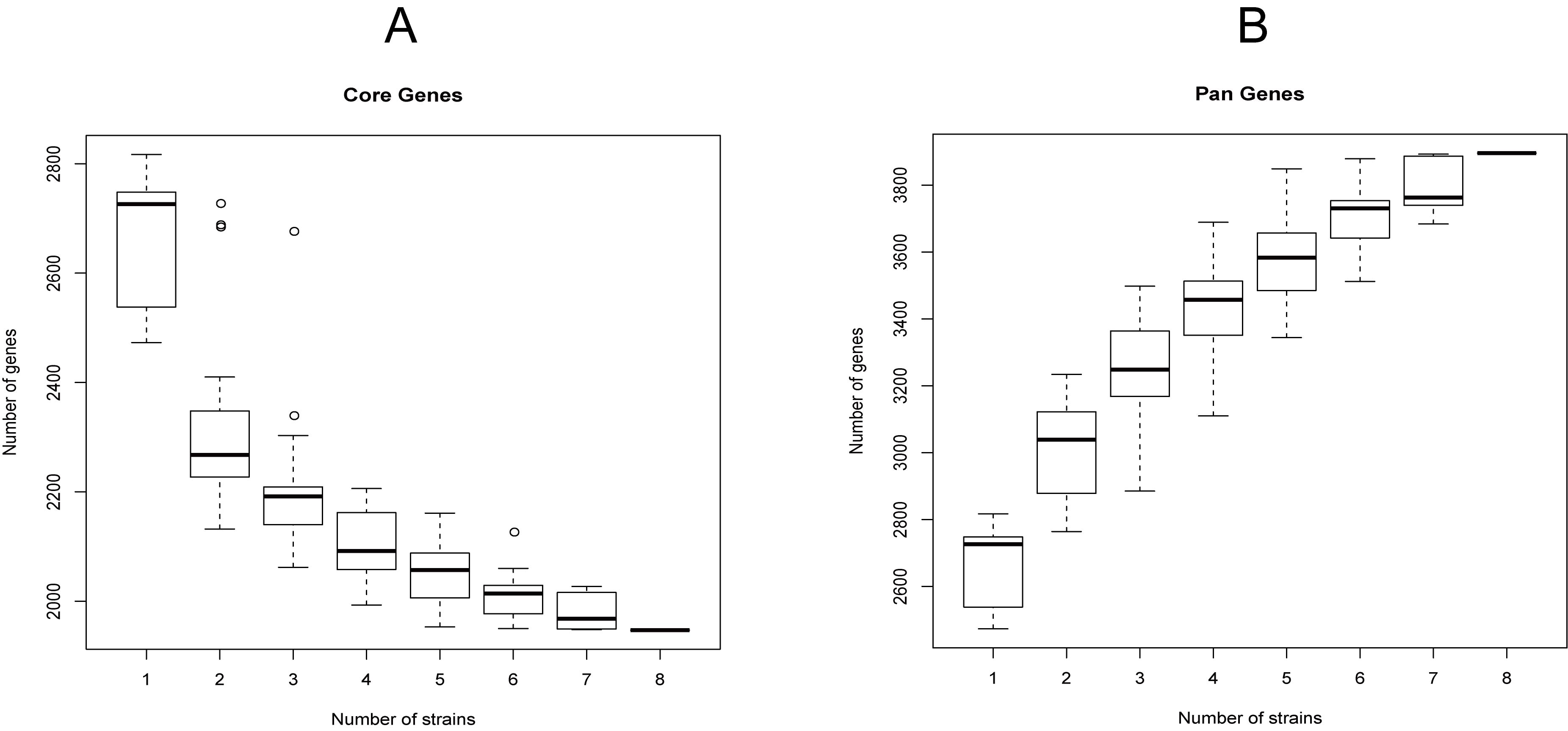

Supplement: Supplementary file 4 [file Image_4.JPEG]

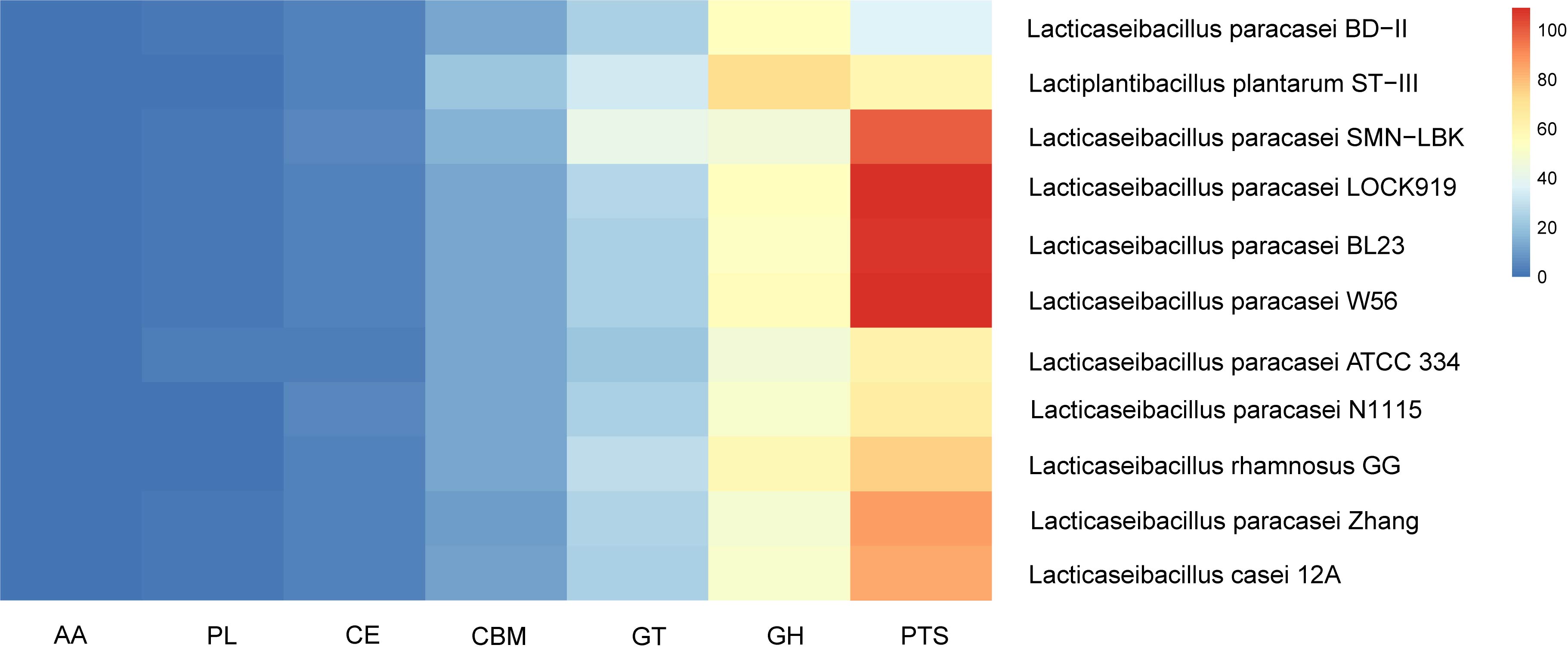

Supplement: Supplementary file 5 [file Image_5.JPEG]
